# A genome wide study of genetic adaptation to high altitude in feral Andean Horses of the páramo

**DOI:** 10.1186/1471-2148-13-273

**Published:** 2013-12-17

**Authors:** Sher L Hendrickson

**Affiliations:** 1 Department of Biology, Shepherd University, Shepherdstown WV 25443, USA

**Keywords:** hypoxia, *Equus*, Hypoxia Inducing Factor, Adaptation, Genomes wide association

## Abstract

**Background:**

Life at high altitude results in physiological and metabolic challenges that put strong evolutionary pressure on performance due to oxidative stress, UV radiation and other factors dependent on the natural history of the species. To look for genes involved in altitude adaptation in a large herbivore, this study explored genome differentiation between a feral population of Andean horses introduced by the Spanish in the 1500s to the high Andes and their Iberian breed relatives.

**Results:**

Using allelic genetic models and Fst analyses of ~50 K single nucleotide polymorphisms (SNPs) across the horse genome, 131 candidate genes for altitude adaptation were revealed (Bonferoni of *p* ≤ 2 × 10^–7^). Significant signals included the *EPAS1* in the hypoxia-induction-pathway (HIF) that was previously discovered in human studies (*p* = 9.27 × 10^-8^); validating the approach and emphasizing the importance of this gene to hypoxia adaptation. Strong signals in the cytochrome P450 3A gene family (*p* = 1.5 ×10^-8^) indicate that other factors, such as highly endemic vegetation in altitude environments are also important in adaptation. Signals in tenuerin 2 (*TENM2, p* = 7.9 × 10^-14^) along with several other genes in the nervous system (gene categories representation *p* = 5.1 × 10^-5^) indicate the nervous system is important in altitude adaptation.

**Conclusions:**

In this study of a large introduced herbivore, it becomes apparent that some gene pathways, such as the HIF pathway are universally important for high altitude adaptation in mammals, but several others may be selected upon based on the natural history of a species and the unique ecology of the altitude environment.

## Background

In addition to hypoxia and high levels of ultraviolet radiation, many aspects of high altitude environments put strong evolutionary pressures on resident species. Extreme temperature and humidity fluctuation, highly endemic vegetation and fauna, and other biological factors relative to a species’ natural history, such as length or timing of the breeding season or level of population isolation can create strong adaptive pressure. Understanding patterns and timing of genetic adaptations through organisms of different life histories is important to elucidate the commonalities in unique adaptation pathways that occur in extreme environments.

The feral horses of the high Andean pàramo were originally brought with the Spanish conquistadors in the 1500s. Although most herds have been extirpated, small groups now called the párameros or cerreros persist in Ecuador’s eastern range between the Cotopaxi volcano and Quilindaña. These horses came predominantly from Andalusia, and had a mixture of Jennet, Andalusia, and Berber ancestry. They quickly adapted to the Andes, interbreeding naturally in small bands in isolation [1], with the consequence that they present an ideal natural experiment in adaptation to high altitude conditions.

In addition to hypoxia, the highly inclement pàramo presents other physiologic and metabolic challenges. The pàramo is cold and humid with yearly rainfalls between 500 to over 3000 mm. Extreme temperature fluctuations, with diurnal ranges from below freezing to up to 30C and periods of fog, heavy rain, hail or even snow dominate a good portion of the day during the rainy season. The dry season is very short. Regular subzero temperatures, high UV radiation, and low pH soils have selected for an highly endemic alpine vegetation primarily consisting of tussock grasses, ground rosettes, dwarf shrubs, cushion plants and giant rosettes such as *Espeletia* and *Puya *[2]. Although *Equus* fossils have been found throughout the Andes, all New World Equids went extinct in the Americas at the end of the Pleistocene [3] and in the high Andes, the remaining large ungulates (i.e. Camelids) shifted their center of distribution to the more temperate puna grasslands to the south [4]. The success of the introduced horses in the northern pàramo provides a unique model for looking at extreme hypoxic, thermoregulatory and metabolic adaptive pressure over a relatively short time span of ~200 generations.

Several recent genome wide studies have focused on human adaptation to hypoxia. Comparisons of high-altitude Tibetan populations with low-altitude Han Chinese populations uncovered strong signatures of selection in the hypoxia inducing factor (HIF) response pathway in the region of endothelial PAS domain 1 (*EPAS1*) [5-7]. Several other genes in this pathway were also associated including peroxi-some proliferator-activated receptor-a (*PPARA*) (Tibetans) and egl 9 homolog 1 (*EGLN1*) (Tibetans and Andeans [5-9]), and the protein kinase, AMP-activated a 1 catalytic subunit (*PRKAA1*) (Andeans [10,11]). Selection in the HIF pathway is detected in most human studies; however, several other pathways such as methylation, pathogen resistance, or DNA damage repair do not clearly overlap between study groups [12]. This could reflect differences in evolutionary paths, time scale, or other natural history variables that have not been elucidated yet.

As a large-bodied herbivore, the introduced Andean horse provides a unique comparative perspective for further understanding whether the HIF pathway is a universal target of natural selection for hypoxia, and how time, altitudinal ecosystem, and species life history influence genetic adaptation to extreme environments. This study explores which genomic regions appear to be under selection in the Andean horse, and how these genes relate to previous studies and to the natural history of the horse and the páramo.

## Results and discussion

### Population structure

The feral population of horses is made up of several small bands located on lands adjacent to the Cotopaxi National Park, including Hacienda Yanahurco, a private wildlife reserve of ~7300 acres, and other unpopulated land to the south. The populations size fluctuates from ~100 to over 200 individuals due to extreme weather fluctuations and removal of animals by wranglers.

**Figure 1:**
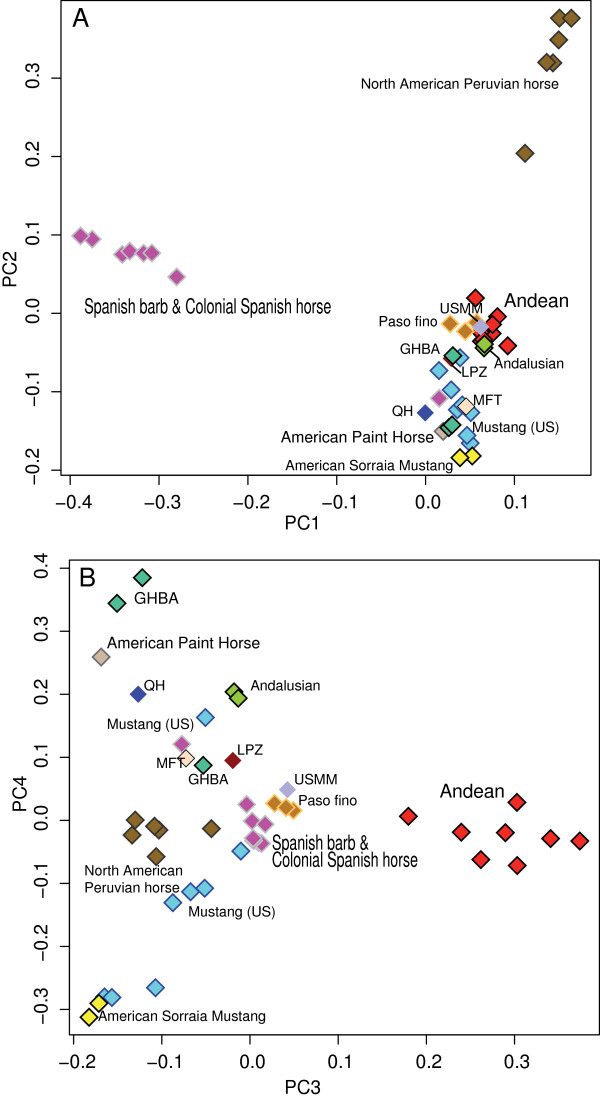
**Principal components analysis of ~54 K SNPs for the Andean feral herd and Iberian and European breeds. A**. Shows Andean horses and Iberian-origin breeds in the Americas cluster with the Andeans close to Paso finos and the US Mangalarga Marchador (USMM). The Colonial Spanish horses (CSH) and Spanish Barbs (SBBA) on the right on PC2 show a tight relationship, possibly related to the fact that several are known to be either from or descents of the Wilber-Cruce herd. **B**. The Andean population forms a tight cluster defined by PC3. Other breeds shown include Mustang (US Spanish Mustang Registry and the American Heritage Horse), Galacino (GHBA), Quarter horse (QH); American Paint Horse; Missouri Fox Trotter (MFT), Lipizzaner (LPZ), Sorraia (some cross listed with Horse of the Americas and the American Sorraia Horse Association), and Paso finos both of the North American Peruvian Horse (NAPHA) variety and from the Paso fino horse association.

Horses are small in stature and many individuals exhibit the heritable “gaited” trait found in breeds such as the Paso fino or Mangalarga Marchador. A principal component analysis indicated that the feral horses form a cluster close to these two breeds in PC1 (Figure 1A), and they are a distinct cluster as defined by PC3 (Figure 1B). Several breeds form tight clusters (e.g. Spanish Barb Breed Association, North American Peruvian horses, Galicinos), indicating either under strong artificial selection and/or a small number of founders. The general arrangement of the clusters reflected relationships illustrated in a larger prior analysis of breeds [13].

### Genome wide allelic differentiation

**Figure 2:**
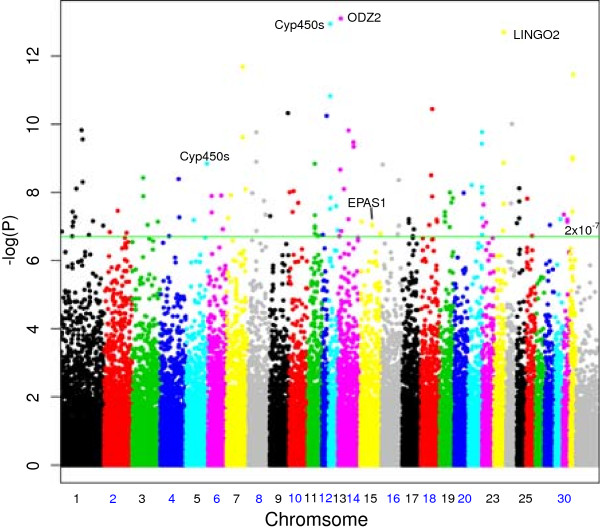
**Plot of chromosomal position verses the –log ( *p *-value) for the frequency divergence between feral Andean horses and the comparison group.** The green line represents a Bonferoni cut-off of *p* < 2 × 10^-7^.

Using Allelic Genetic models for association, 129 SNPs in the feral Andean horses show genetic divergence from the mixed breed sample below the Bonferoni cut-off (*p* < 2 × 10^-7^) as shown in the Manhattan plot in Figure 2 (*p*-values given in Additional file 1: Table S2). 131 genes are located within the regions identified by these SNPs that may have been under strong selection once horses were introduced into the Andes. Table 1 lists the most significant ten associations, and each region of interest is discussed in turn below.

**Table 1:** Most significant allelic divergences between feral Andean horses and breed out-groups

**Rank *p*-value**	**Chr**	**SNP**	**Position**	**Allele 1**	**Allele 2**	**P**	**Fst**	**block**	**gene**
1	14	BIEC2-245079	12656702	A	G	7.90 × 10^-14^	Y	12,646,702-12,746,386	TENM2*
2	14	BIEC2-245080	12656714	A	G	7.90 × 10^-14^			TENM2*
	13	BIEC2-207449	7283092	G	A	3.01 × 10^-8^	Y	6,917,885-7,474,810	CYP3A93*
3	13	BIEC2-207503	7359845	G	A	1.14 × 10^-13^			CYP3A89
7	13	BIEC2-207537	7369733	G	A	1.48 × 10^-11^			CYP3A94-97
4	23	BIEC2-625752	45817096	G	A	2.02 × 10^-13^	Y	45,807,096-45,855,318	LINGO2*
	23	BIEC2-625758	45845236	A	C	2.19 × 10^-8^			LINGO2*
	23	BIEC2-625761	45845318	A	G	1.36 × 10^-9^			LINGO2*
5	7	BIEC2^-^1007064	75718493	G	A	2.08 × 10^-12^		75,708,493-75,744,878	
	7	BIEC2-1007074	75744878	C	A	2.42 × 10^-10^			
6	31	BIEC2-837371	13819214	C	A	3.48 × 10^-12^		13,793,328-13,819,214	
	18	BIEC2-412366	49758616	A	G	1.33 × 10^-8^	Y	49,492,862-50,094,655	MYO3B*
8	18	BIEC2-412389	50094655	A	G	3.59 × 10^-11^			UBR3, Sp5
9	9	BIEC2-1106047	77359939	G	A	4.73 × 10^-11^		77,356,508-77,367,776	
10	12	BIEC2-194315	23226819	A	G	5.67 × 10^-11^		23,216,819-23,236,819	SLC22A11

A “*” indicates that the SNP listed is within the gene.

### TENM2 and other nervous system genes

**Figure 3:**
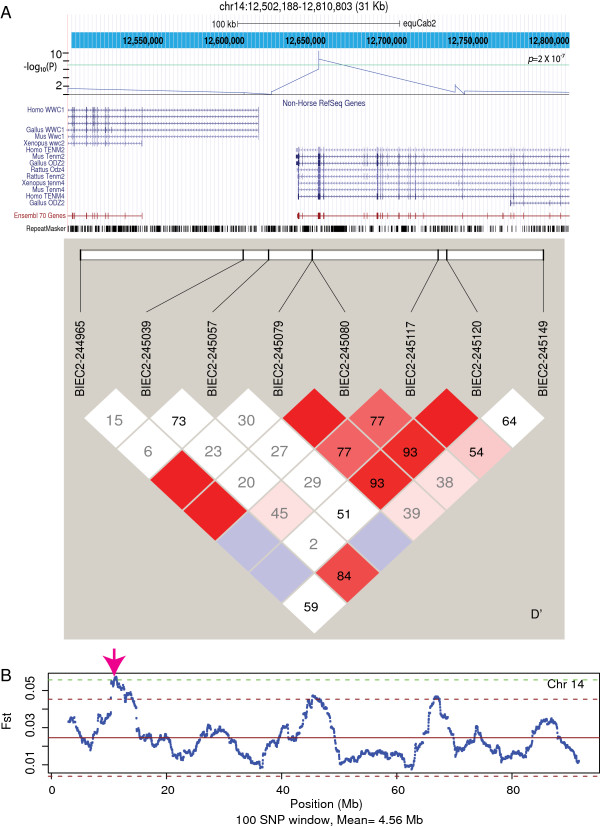
**The most significant allelic frequency divergence found in this study between feral Andean horses and the comparison group within the *TENM2 *gene. A** The arrangement of TENM2 genes from different species is shown from the UCSC browser along with the –log (*p*-value) for individual SNPs in the region. The green line represents genome wide significance. Linkage disequilibrium between adjacent SNPs (D’) is shown the browser graph. **B** shows the Fst nalysis of Chromosome 14 with the region of TEMN2 indicated (arrow). The green dotted line represents 2 standard deviations from the mean Fst value for chromosome 14.

The most significant divergence in allele frequencies in the Andean horse is found in the *TENM2* gene, a member of the teneurin family of type II tramsmembrane glycoproteins (*p* = 7.9 × 10^–14^, Figure 3). This signal is also supported by Fst analysis (Figure 3B). The genes for teneurins are highly conserved across invertebrate and vertebrate species. The region on chromosome 14 in *Equus* shares 93.7% sequence identity with human *TENM2*, and 81.7% with chicken *TENM2* (formerly *ODZ2*).

Teneurin proteins are proposed to regulate gene expression in the nervous system during development [14]. *TEMN2* is involved in development of neuronal circuits in the visual system [15] and has been shown to be expressed in the developing limbs, somites, and craniofacial mesenchyme [16].

Further support for the nervous system as a focus of adaptation in the feral Andean horse population is that "*neurological system process*" was the most significantly represented category in a Gene Ontology Biological Processes analysis (GO:0050877, *p* = 5.1 × 10^-5^) (Additional file 2: Table S3). Eighteen of 87 genes categorized in GO fell into this category (Table 2), including *EPAS1*. Hypoxia is implicated in several human central nervous system pathologies such as stroke or neurodegenerative disease and a connection between the HIF pathway and neuronal response to hypoxia has been recognized [17]. 12 of the genes were in sensory perception, particularly olfaction, which may indicate that better sensory abilities are a benefit in the wild where individuals must locate mates, maintain herd structure, avoid predators and find appropriate food sources. In addition developmental and functional genes within the nervous system were significant.

**Table 2:** **Neurological system process genes represented in a Gene ontology biological processes analysis (GO:0050877, *p*** **= 5.1 × 10**^**-5**^**)**

**Entrez gene ID**	**Gene**	**Gene name**
114902	C1QTNF5	C1q and tumor necrosis factor related protein 5
6558	SLC12A2	solute carrier family 12 (sodium/potassium/chloride transporters), member 2
2034	EPAS1	endothelial PAS domain protein 1
30820	KCNIP1	Kv channel interacting protein 1
8013	NR4A3	nuclear receptor subfamily 4, group A, member 3
2895	GRID2	glutamate receptor, ionotropic, delta 2
130507	UBR3	ubiquitin protein ligase E3 component n-recognin 3 (putative)
1756	DMD	dystrophin
117194	MRGPRX2	MAS-related GPR, member X2
138799	OR13C5	olfactory receptor, family 13, subfamily C, member 5
138805	OR13F1	olfactory receptor, family 13, subfamily F, member 1
138804	OR13C4	olfactory receptor, family 13, subfamily C, member 4
138803	OR13C3	olfactory receptor, family 13, subfamily C, member 3
140469	MYO3B	myosin IIIB
138802	OR13C8	olfactory receptor, family 13, subfamily C, member 8
392376	OR13C2	olfactory receptor, family 13, subfamily C, member 2
6854	SYN2	synapsin II
390061	OR51Q1	olfactory receptor, family 51, subfamily Q, member 1

The genes in this category are listed below. Analysis was done with the human background that is more complete. ENTREZ gene IDs given are for *Homo sapiens.*

### Cytochrome P450 genes

**Figure 4:**
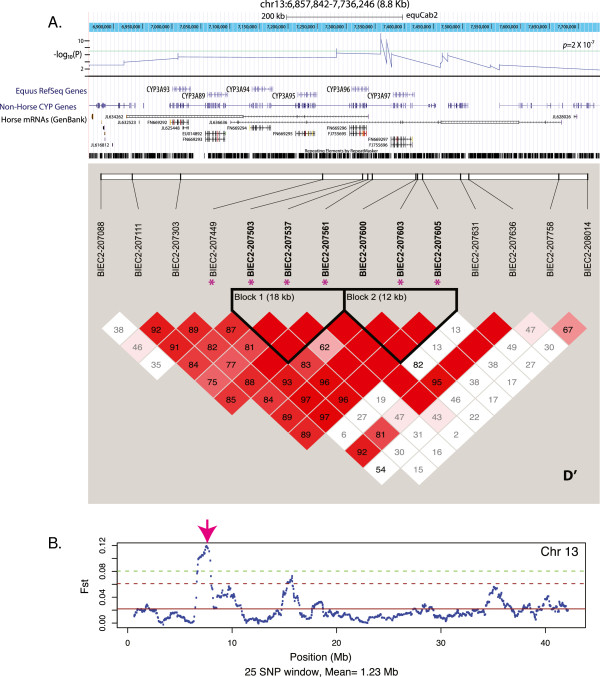
**The placement of significant SNPs within the horse cytochrome P 450 genes. A**. The horse CYP3A93-96 genes align to CYP3A5 (human and several other species, Bos CYP3A4) and downstream of the horse cluster, CYP3A6. The –logP for individual SNPs in the region is indicted. The green line represents genome wide significance. Linkage disequilibrium as D’ is shown. **B**. Shows the Fst nalysis of Chromosome 13 with the region of CYP3A indicated (arrow). The green dotted line represents 2 standard deviations from the mean Fst value for chromosome 13.

Significant frequency divergences occur on chromosome 13 in a region containing several cytochrome P450 (CYP) 3A genes (Figure 4). Three SNPs show significant allele frequency divergence meeting the Bonferoni correction of p <2 × 10^-7^ and three additional SNPS meet a lower cut-off of 10^-6^ (Bonferoni of p < 0.05, BIEC2-207561, BIEC2-207603, BIEC2-207605). The SNP BIEC2-207449 is located within *CYP3A93*; however the entire horse *CYP3A* gene cluster consists of seven genes and one pseudogene in strong linkage disequilibrium covering a region of ~115 Kb. Fst analyses also supported this region as a candidate of selection (Figure 4B).

Cytochrome P450 enzymes are a superfamily of membrane bound heme containing monooxygenases whose function is to catalyze the oxidation of organic substances. Best known for their role in drug metabolism, CYPs also are involved in hormone synthesis and breakdown, cholesterol synthesis, vitamin D metabolism, and metabolism of toxic compounds. The horse CYP3A gene cluster is more extensive than the human CYP cluster. It has been suggested that the horse CYP gene cluster is more complex because horses must digest a range of plant toxins in different habitats [18]. In the páramo ~ 60% of plant species are endemic and include largely bunchgrasses, shrubs, mosses and lichens which are highly evolved, likely in response to high UV, low pH soil and temperature and humidity fluctuation. This vegetation would differ greatly from that originally found in the habitats of the founding horses. It should be noted that members of this gene family were found in genome-wide hypoxia adaptation studies of Tibetan human populations [5] and frogs [19] and experimental data from rabbits indicate hypoxemia up-regulates the expression of CYP3A6 [20].

### Replication of associations between EPAS1 and life at high altitude

**Figure 5:**
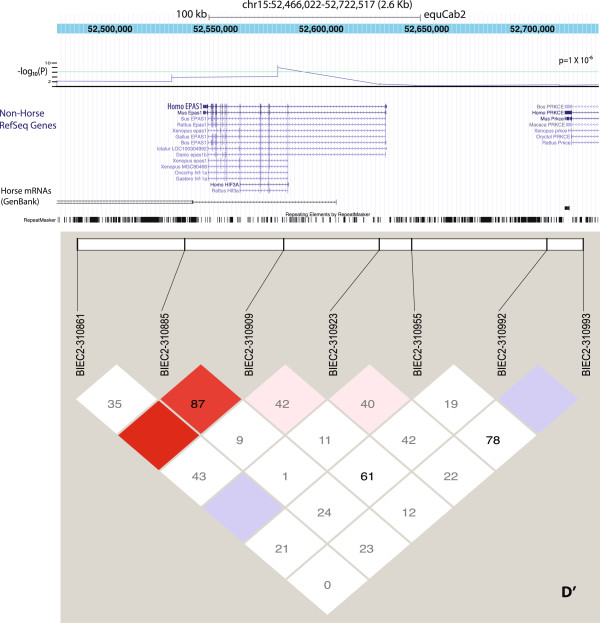
**The placement of significant SNPs within the EPAS1 gene**. The –log(*p*-value) for individual SNPs in the region is indicted above the assembly of EPAS1 genes. The green line represents genome wide significance. D’ values are shown. The SNP shown, BIEC2–310909 (rs69041973) was highly significant (*p* = 9.27 × 10^-8^). This SNP is an intronic SNP with no known function.

EPAS1 has been found to be associated with altitude in several studies of Tibetans [5-7,21]. EPAS1 encodes a transcription factor involved in the induction of genes regulated by oxygen, which is induced as oxygen levels fall. In the horse, BIEC2–310909 (rs69041973) was highly significant (*p* = 9.27 × 10^-8^) as shown in Figure 5. This SNP is an intronic SNP with no known function. It has been suggested that in Tibetans, the association between EPAS1 expression and lower Hb levels is an adaptive response that lowers hypoxia-induced erythropoeisis in Tibetans such that the negative effects of this hypoxia response do not occur [6,22]. A more in-depth follow-up study will be needed to confirm the result and elucidate the exact mechanism for the difference, but the commonality of this gene association in horses with the studies in humans is intriguing.

### Other signals

Other genes with high allele frequency differences within the Andean herd include *LINGO2*, which was recently associated with BMI [23] and Parkinson disease [24,25], and a region containing ubiquitin protein ligase E3 component n-recognin 3 (*UBR3*), Sp5 transcription factor, glutamate decarboxylase 1 (*GAD1*) and myosin IIIB (*MYO3B*). Solute carrier family 22 (organic anion/urate transporter), member 11 (*SLC22A11*) (*p* = 5.7 × 10^–8^) is part of the SLC22A gene family that mediates the absorption, distribution, and excretion of a diverse array of environmental toxins [26].

**Figure 6:**
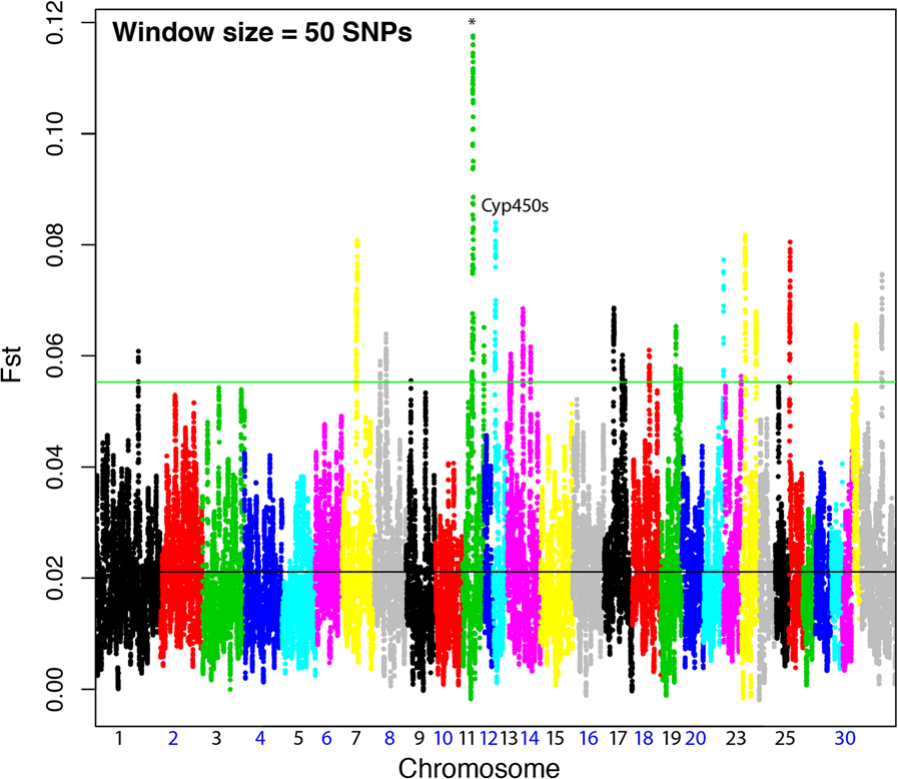
**FST analysis results across the feral Andean horse genome in comparison to a mixed breed outgroupc.** The top peaks are labeled with genes in the region when possible. The highest signal on Chromosome 11 contains several genes, TOM1L1, COX11, STXBP4, HLF, MMD, TMEM100, and PCTP. The Fst data was corroborated by a highly significant association found between COX11/STXBP4, which is perhaps the most biologically interesting.

Most Fst signals reflected the significant association signals. The highest region of allele frequency divergence as measured by Fst was observed on chromosome 11 (Figure 6), and contained the following genes: *TOM1L1*, *COX11*, *STXBP4*, *HLF*, *MMD*, *TMEM100*, and *PCTP*. The Fst data was corroborated by a highly significant association found between *COX11*/*STXBP4* (*p* = 1.44 × 10^-9^). These two genes have been explored for their association to human breast cancer in several studies [27-30]. They are in strong LD in both humans and horses. *COX11* is a nuclear encoded mitochondrial protein and the terminal component of the mitochondrial respiratory chain. *COX11* has been found to be up-regulated in chronic hypoxia of cyanotic patients undergoing repair of heart defects, suggesting a role in dealing with lack of oxygen, possibly by acting as an heme biosynthetic enzyme which transports copper to heme A [31,32].

## Conclusions

Several large allelic divergences between the feral Andean horses and a comparison group of primarily Iberian horse breeds have been observed in this study. Many of the genes identified in this study appear to be biologically relevant, however one cannot rule out type 1 error due to genetic drift or the composition of the comparison group. A recent study of artificial selection in 33 horse breeds found strong selection for aesthetics and performance traits resulted in high homozygosity within breeds and large divergence between breeds [33]. The Andean population was likely founded by a mixed sample of Iberian breeds; however, by combining the descendants of those breeds in the comparison group, many of which have undergone strong subsequent artificial selection since their introduction to the Americas, one effectively makes a comparison to a more heterogeneous out-group. This may increase the potential for false positives. The replication of the significance of the *EPAS1* loci suggests that many genes found in this study are the result of strong selection in the wild population. The replication of this signal in a large herbivore in a relatively short evolutionary time span further implicates HIF- response pathway as essential to high altitude. Additional signals in the nervous system and the cytochrome-P450 gene family, which may be in response to the local endemic plants, suggest that high altitude adaptation is complicated, and that life history of the species in question and local ecology of the environment are both important factors in evolutionary adaptation.

## Methods

### Population samples

Population samples from the feral horse herd (n = 97) were collected on the southeastern side of the Cotopaxi National Park in Ecuador (0°41´28.97¨ S 78°16´44.69¨ W) in November 2006 and March 2007 during the annual “round-up” which was started in the late 1990s. DNA in this study is from pulled hair because it is non-evasive and can be collected rapidly; however, blood and small skin clippings were collected when possible and preserved in RNALater (Ambion, Inc). No samples were taken from foals born during the current season to avoid excess stress. The round-up employs Chagras from adjacent villages who ride horses originally captured from the feral herd in previous years, therefore samples were also obtained from riding and pack horses.

For a comparison group, registered breed samples (N = 55) were collected in the United States and South America primarily from Iberian breeds (Andalusian, Lipizzaner) or breeds that have recently been defined and founded in the Americas from original Spanish-mixed stock; including Colonial Spanish Horse, Sorraia Mustang, American Paint Horse, American Heritage Horse, Horse of the Americas, Galiceno, North American Peruvian Horse, Paso Fino, Quarter Horse, US Mangalarga Marchador and Spanish Barb (details given in Additional file 3: Table S1). More distant breed samples included Arabian (Bask and Polish), Shetland Pony, Miniature Horse, Thoroughbred, and Sandohit Oldenburg. The Andean populations were likely founded from horses from more than one Iberian breed. Further, a recent study of artificial selection showed that many breeds included in this study have been under strong selection for aesthetic and performance traits resulting in high level of homogeneity within breeds [33]. Artificial selection would have continued after the Iberian breeds were established in the Americas. Therefore breeds were combined to lesson artifacts from artificial selection and to provide a more heterogeneous out-group similar to the potential founding population.

### DNA isolation and genotyping

DNA was isolated with the Qiagen DNeasy Blood and Tissue Kit (Valencia, CA) following the manufacturer’s protocol with the exception of over-night digestion at 56C for all samples. The Equine SNP50 BeadChip (Illumina, Inc., San Diego, CA) was used to genotype 50,023 SNPs across the horse genome in 151 horses from the Andean population and related breeds. Average spacing between functional SNPs on the chip is 43.1kB with lower coverage on chromosome X (49.44kB) [34]. The average genotyping completeness in this study was 99.7% (92.0–99.9%).

Initial quality control (QC) was performed in Genome Studio version 2010.3 (Illumina, Inc., San Diego, CA). Of the 54,602 SNPs typed, 2200 were excluded due to <60% Gentrain Scores (n = 917), <95% Call frequency (n = 625), or >3 clusters or low intensity scores (n = 657). No SNPs had >2 Mendel Errors based on the 3 trios included in the genotyping. 108 SNPs were excluded with missingness >5% between case and controls. Of the remaining 52,294, 50,023 had a minor allele frequency of >0.01 and were included in subsequent analyses. Of these, 584 (0.01%) were monomorphic within Andeans.

### Estimations of relatedness and population structure

Because the samples in this study were collected during a round–up of an unmarked herd over a large montane area, it was necessary to estimate relatedness between samples, and then remove all first order relatives from population analyses. Kinship coefficients were based on 33,483 SNPs pruned to remove those in high linkage disequilibrium (independent pairwise pruning, window size = 50, r^2^ = 0.5) in PLINK version 1.07 [35]. 18 feral individuals had a kinship coefficient > 0.5, of which 8 individuals were removed from subsequent analyses. Kinship coefficients also identified 9 offspring of an Arabian stallion introduced to the riding herd adjacent to the hacienda in 2006, which were not included in downstream analyses. Kinship coefficient distributions from the samples collected indicate the population is not extremely inbred (Additional file 4: Figure S1).

The population structure of the feral Andean horses and several related breeds were analyzed by principle component analyses in Eigensoft 3.0 [36] with LD-pruned dataset (32, 672 SNPs). These data confirmed the half Arabian offspring as well 6 horses originally from the wild herd that were now owned by local Chagras.

### Genome–wide statistical analyses

Selection was analyzed on two levels. First a SNP-by-SNP allelic model was performed in PLINK (version 1.07) [35] between feral Andean horses (n = 78) and the comparison group to look for significant differences in allele frequency between the horses that have been living at altitude (3500-4500 m) for the past 500 years and founding breeds. P–values were based on the Fisher’s Exact test. Allelic models for all SNPs in the analyses are shown in Additional file 5. A Bonferroni correction of p < 2 × 10^–7^ used to define genome-wide significance.

A second analysis using sliding window Fst was applied to look for signals of selection [37] within each chromosome with window sizes of 100, 50 and 25 SNPs, which were on average 4.7 ± 0.2, 2.3 ±0.2 and 1.2 ±0.2 Mb in size respectively.

### Gene identification

Genes within statistically significant regions were identified in the *Equus* assembly equCab2.0 (2007) within the UCSC browser [38,39]. Genes were included if they were within ±10Kb from the significant SNP. In cases where large blocks of LD surrounded the SNP, or several adjacent SNPs were significant, these areas were included in for gene identification. Human and other available reference assemblies were aligned to the horse assembly to assist in identification of candidate genes. A GO functional analysis was performed on the genes found with the GWAS method with Database for Annotation, Visualization and Integrated Discovery (DAVID) v6.7 [40]. P-values were based on the Fisher’s Exact test.

### Availability of supporting data

Genotyping data can be found at https://mynotebook.labarchives.com/share/Horse%2520SNP%2520chip%2520/My45fDI0OTgzLzMvVHJlZU5vZGUvMjQ0NjE5MjkxN3w5Ljk=.

## Competing interests

The author declares that she has no competing interests.

## Supplementary Material

Additional file 1: Table S2 SNPs found to be associated with the Andean horse population association using the allelic model at p<0.05 (Bonferoni p<1 ×10^-6^). Additional analyses were limited to p<0.01 (Bonferoni p<2 ×10^-7^). * = SNP located in gene. Click here for file

Additional file 2: Table S3 Gene Ontology Biological Processes analysis. Click here for file

Additional file 3: Table S1 Horse samples included in the study. No.=The number of individuals within that group. Click here for file

Additional file 4 **Kinship coefficients distribution for the Andean horses collected during the study based on 33,483 LD-pruned SNPs (independent pairwise pruning, window size = 50, r**^**2**^** = 0.5, PLINK version 1.07).** Click here for file

Additional file 5: Table S4 All results from association tests between the Andean horse population and the outgroup using the allelic model. Fisher’s p-value. A1=Allele 1, A2= Allele 2. Click here for file
