# Supplementary material for: A genome wide study of genetic adaptation to high altitude in feral Andean Horses of the páramo
Source: BMC Evol Biol. 2013 Dec 17;13:273. doi: 10.1186/1471-2148-13-273 (PMC3878729; doi:10.1186/1471-2148-13-273)
Supplement: Additional file 3: Table S1 — Horse samples included in the study. No.=The number of individuals within that group. [file 1471-2148-13-273-S3.docx]

Supplementary Table 1. Horse samples included in the study. No.=The number of individuals within that group.

| No. | Breed (Registries listed when appropriate) |
| --- | --- |
| 2 | American Heritage Horse Association and Spanish Mustang Registry |
| 1 | American Heritage Horse Association and Spanish Mustang Reg., Horse of the Americas, Southwest Spanish Mustang Association |
| 1 | American Paint Horse Association |
| 1 | American Sulpher Horse Association and Sorraia Mustang Studbook |
| 1 | Andalusian Horse Registration |
| 82 | Andean feral horse |
| 6 | Andean horses originally from feral population (Chagra owned) |
| 8 | Andean horse from local domestic stock (Chagra owned) |
| 1 | Arabian stud introduced to local herd |
| 1 | Arabian (Bask line) |
| 1 | Arabian (Polish-US) |
| 3 | Colonial Spanish Horse |
| 5 | Galacino Horse Breeder Association |
| 1 | Horse of the Americas and Sorraia Mustang Studbook |
| 1 | IALHA-International |
| 1 | Miniature horse |
| 1 | Missouri Fox Trotter Horse Breed Association |
| 10 | North American Peruvian Horse Association |
| 3 | Paso Fino Horse Association |
| 1 | Sandohit Oldenburg (tattooed) |
| 1 | Shetland Pony |
| 1 | Sicilian donkey |
| 4 | Spanish Barb Breeders Association |
| 1 | Spanish Barb Breeders Association, Spanish Mustang Registry |
| 8 | Spanish Mustang Registry |
| 1 | Thoroughbred (tattooed) |
| 1 | United States Lipizzan Registry |
| 1 | US Mangalarga Marchador |
| 2 | Quarter Horse Registry |

151
