# Supplementary figures and images for: A genome wide study of genetic adaptation to high altitude in feral Andean Horses of the páramo
Source: BMC Evol Biol. 2013 Dec 17;13:273. doi: 10.1186/1471-2148-13-273 (PMC3878729; doi:10.1186/1471-2148-13-273)

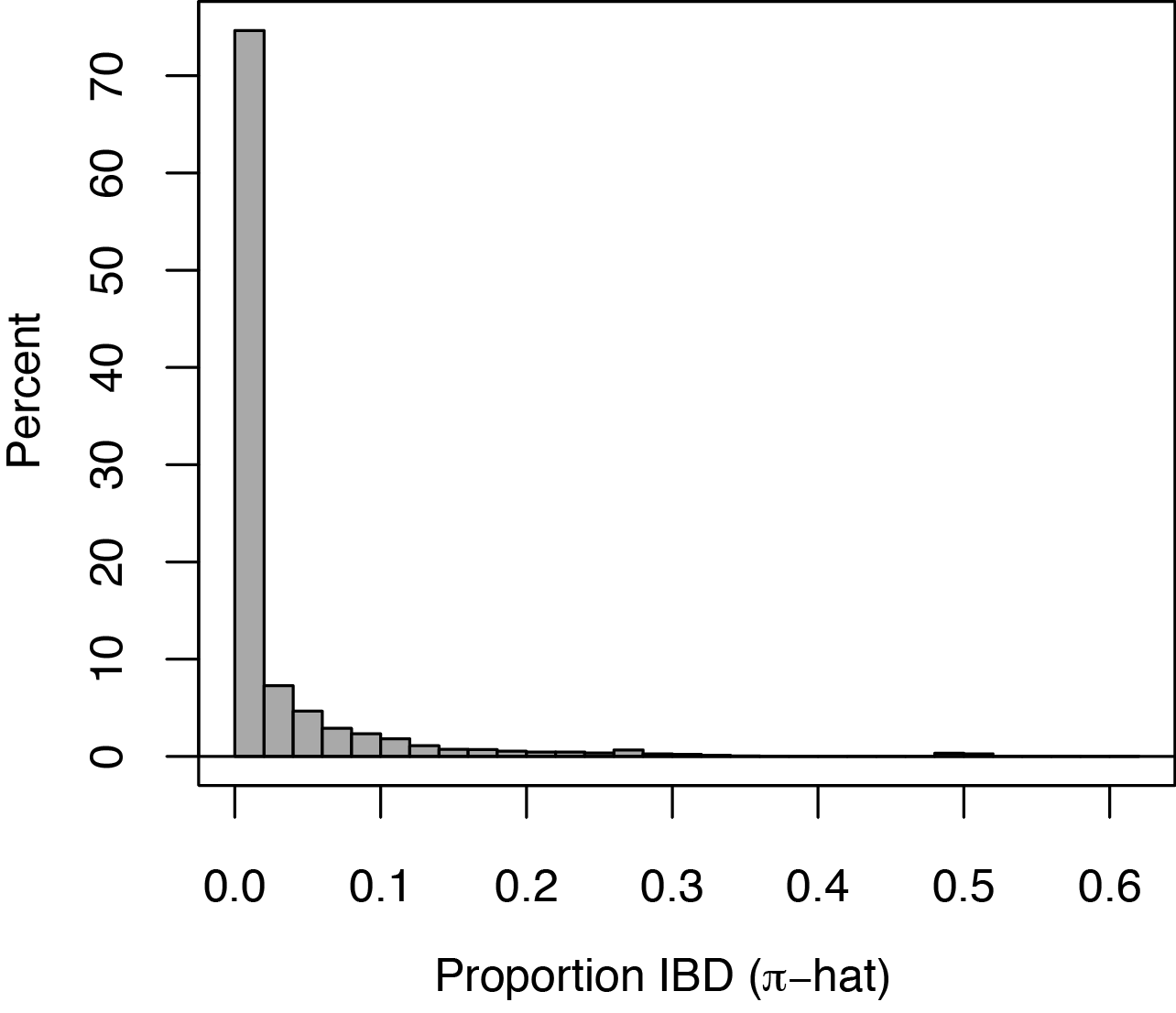

Supplement: Additional file 4 — Kinship coefficients distribution for the Andean horses collected during the study based on 33,483 LD-pruned SNPs (independent pairwise pruning, window size = 50, r 2 = 0.5, PLINK version 1.07). [file 1471-2148-13-273-S4.png]
